# Whole-body diffusion-weighted MRI for staging of women with cancer during pregnancy: a pilot study

**DOI:** 10.1186/1470-7330-15-S1-P50

**Published:** 2015-10-02

**Authors:** RC Dresen, SN Han, K Michielsen, F De Keyzer, MM Gziri, F Amant, V Vandecaveye

**Affiliations:** 1 Department of Radiology, Leuven Cancer Institute, University Hospitals Leuven, KU Leuven, Leuven, Belgium; 2 Department of Obstetrics and Gynaecology, Division of Gynaecologic Oncology, Leuven Cancer Institute, University Hospitals Leuven, KU Leuven, Leuven, Belgium

## Aim

To evaluate whole-body diffusion weighted magnetic resonance imaging (WB-DWI) for staging of women with cancer during pregnancy.

## Methods

Twenty patients diagnosed with cancer during pregnancy underwent WB-DWI additional to conventional imaging in this prospective single centre study. Reproducibility of WB-DWI between 2 readers was evaluated using Cohen’s κ statistics and accuracy was compared to conventional imaging for assessing primary tumour site, nodal metastases and visceral metastases. Histopathology after surgery or biopsy was the primary reference standard.

## Results

Ten patients had breast cancer, 3 lymphoma, 2 cervical uterine cancer, 1 ovarian borderline tumour, 2 colon cancer, 1 lung cancer and 1 a conjunctival tumour. The WB-DWI readers showed very good agreement for lesion detection, κ = 0.94. With WB-DWI, reader 1 detected 38 of 41 malignant lesions, reader 2 thirty-nine lesions and conventional imaging 27. WB-DWI showed sensitivity of 95% (95% CI: 74-99) for both readers and specificity up to 99% (95% CI: 76-99) compared to 50% sensitivity (95% CI: 28-72) with 100% (95% CI: 97-100) specificity for conventional imaging. For staging distant metastases, WB-DWI sensitivities were 66.7% (95% CI: 13-98) and 100% (95% CI: 40-100) respectively for reader 1 and 2 with specificities of 94.1% (95% CI: 69-99) and 100% (95% CI: 40-100) compared to sensitivity of 33.3% (95% CI: 1.7-87) and specificity of 100% (95% CI: 77-100) for conventional imaging.

## Conclusion

WB-DWI is feasible for single-step non-invasive imaging based cancer staging during pregnancy showing additional value to conventional imaging procedures for detecting distant and nodal metastases.

